# Ultrasound assessment of ovarian cancer risk in postmenopausal women with CA125 elevation

**DOI:** 10.1038/sj.bjc.6690575

**Published:** 1999-07

**Authors:** U Menon, A Talaat, A R Jeyarajah, A N Rosenthal, N D MacDonald, S J Skates, K Sibley, D H Oram, I J Jacobs

**Affiliations:** Gynaecology Cancer Research Unit, St. Bartholomew's and The Royal London School of Medicine and Dentistry, London EC1A 7BE, UK; Department of Medicine, Harvard Medical School and Massachusetts General Hospital, Boston, MA 02114-2521, USA

**Keywords:** ovarian cancer, risk, ultrasound, CA125, screening

## Abstract

We have previously shown that, in asymptomatic post-menopausal women, serum CA125 elevation is associated with a 36-fold increase in risk of ovarian cancer. This study was undertaken to assess the value of pelvic ultrasound for further stratification of ovarian cancer risk. Of 22 000 post-menopausal women, aged ≥ 45 participating in an Ovarian Cancer Screening Trial, 741 with a CA125 ≥ 30 U ml^−1^ underwent pelvic ultrasonography. Twenty index cancers (primary invasive epithelial carcinomas of the ovary and fallopian tube) were diagnosed amongst these 741 women during a median follow-up of 6.8 years. Ultrasound results separated the women with CA125 elevation into two groups. Those with normal ovarian morphology had a cumulative risk (CR) of index cancer of 0.15% (95% confidence interval (CI) 0.02–1.12) which is similar to that of the entire population of 22 000 women (0.22%, 95% CI 0.18–0.30). In contrast, women with abnormal ovarian morphology had a CR of 24% (15–37) and a significantly increased relative risk (RR) of 327 (156–683). Ultrasound can effectively separate post-menopausal women with raised CA125 levels into those with normal scan findings who are not at increased risk of index cancer and those with abnormal findings who are at substantially increased risk of index cancer. © 1999 Cancer Research Campaign


*British Journal of Cancer* (1999) **80**(10), 1644–1647
© 1999 Cancer Research Campaign
Article no. bjoc.1999.0575

### Ultrasound assessment of ovarian cancer risk in postmenopausal women with CA125 elevation

**U Menon[1], A Talaat[1], AR Jeyarajah[1], AN Rosenthal[1], ND MacDonald[1], SJ Skates[2], K Sibley[1], DH Oram[1] and IJ Jacobs[1]**

[1]Gynaecology Cancer Research Unit, St. Bartholomew's and The Royal London School of Medicine and Dentistry, London EC1A 7BE, UK; [2]Department of Medicine, Harvard Medical School and Massachusetts General Hospital, Boston, MA 02114-2521, USA

**Summary** We have previously shown that, in asymptomatic post-menopausal women, serum CA125 elevation is associated with a 36-fold increase in risk of ovarian cancer. This study was undertaken to assess the value of pelvic ultrasound for further stratification of ovarian cancer risk. Of 22 000 post-menopausal women, aged ≥ 45 participating in an Ovarian Cancer Screening Trial, 741 with a CA125 ≥ 30 U ml$^{-1}$ underwent pelvic ultrasonography. Twenty index cancers (primary invasive epithelial carcinomas of the ovary and fallopian tube) were diagnosed amongst these 741 women during a median follow-up of 6.8 years. Ultrasound results separated the women with CA125 elevation into two groups. Those with normal ovarian morphology had a cumulative risk (CR) of index cancer of 0.15% (95% confidence interval (CI) 0.02–1.12) which is similar to that of the entire population of 22 000 women (0.22%, 95% CI 0.18–0.30). In contrast, women with abnormal ovarian morphology had a CR of 24% (15–37) and a significantly increased relative risk (RR) of 327 (156–683). Ultrasound can effectively separate post-menopausal women with raised CA125 levels into those with normal scan findings who are not at increased risk of index cancer and those with abnormal findings who are at substantially increased risk of index cancer.

**Keywords:** ovarian cancer; risk; ultrasound; CA125; screening

The CA125 tumour marker has an established clinical role in monitoring the course of ovarian cancer during and after therapy (Rustin et al, 1996*a*, 1996*b*). In addition this marker is of value as a prognostic indicator (Gadducci et al, 1995; Nagele et al, 1995) and in the differential diagnosis of pelvic adnexal masses in symptomatic patients (Jacobs et al, 1990; Tingulstad et al, 1996). CA125 is also currently under investigation as a potential screening test for ovarian cancer (Jacobs et al, 1993). Although the role of CA125 in screening remains controversial, there is evidence that serum elevation is associated with an increased risk of ovarian cancer. We have previously reported that asymptomatic postmenopausal women with a CA125 ≥ 30 U ml$^{-1}$ have a 36-fold increased risk of diagnosis for ovarian cancer in the subsequent year (Jacobs et al, 1996). This data was derived from a multimodal screening study which used pelvic ultrasound as a secondary test. We have now been able to perform a further analysis to quantify the value of pelvic ultrasound in refining the risk of cancer amongst asymptomatic women with CA125 elevation.

### METHODS

### Design

The overall study design has been described in a previous report (Jacobs et al, 1988, 1993). A total of 22 000 post-menopausal women, aged ≥ 45 years underwent serum CA125 screening. Women with a CA125 level ≥ 30 U ml$^{-1}$ underwent an ultrasound. Transabdominal and/or transvaginal ultrasonography was used to measure the diameter of each ovary in three planes and to document ovarian morphology. Ovarian volume was calculated using the formula for an ovoid (Campbell et al, 1989). Ovarian morphology was classified as normal if the ovaries exhibited uniform hypoechogenicity and smooth outlines. All other morphological appearances were classified as abnormal. Women with abnormal results on scan were referred to a gynaecologist for assessment and further management. All women were followed up annually during the study by a questionnaire that specifically enquired about any illness or hospital visit in the previous year. When information suggested that a study participant may have had a gynaecological malignancy, histopathological and clinical information was obtained from the general practitioner and/or hospital.

### Statistical analysis

Index cancers were defined as primary invasive epithelial carcinomas of the ovary and fallopian tube (Jacobs et al, 1996). The risk of index cancer at time interval $t$ from the date of ultrasound in volunteers with scans satisfying a particular ultrasound criterion (abnormal ovarian morphology and volume over a specified cut-off) was calculated by dividing the number of volunteers with scans satisfying that criterion who developed index cancers within time $t$ by the total number of volunteers with scans satisfying the criterion. Cumulative risk curves were constructed by plotting the calculated risk values against time. The observed risk of index cancer for all 22 000 study volunteers as well as for all volunteers with CA125 ≥ 30 U ml$^{-1}$ at a given time interval $t$ had been previously calculated (Jacobs et al, 1996). The relative risks of cancer at 1 and 5 years were computed by dividing the calculated risk for women satisfying a specified ultrasound criterion at 1 and 5 years by the observed risk for the entire study population at 1 and 5 years respectively. Confidence intervals (CI) for the risk estimates and

*Received 28 September 1998*
*Revised 12 January 1999*
*Accepted 27 January 1999*

*Correspondence to:* IJ Jacobs

**Table 1** Relative risk of index cancer at 1 and 5 years follow-up for various ovarian ultrasound criteria

| Criteria | USS | Relative risk[a] 1 year | Relative risk 5 years | Serum CA125 (U/ml) Median (range) |
|---|---|---|---|---|
| Entire population[b] | | 1 | 1 | |
| CA125 < 30 U ml$^{-1}$ [b] | | 0.13 (0.03–0.58) | 0.54 (0.32–0.91) | |
| CA125 ≥ 30 U ml$^{-1}$ [b] | | 36 (18–70) | 14.3 (8.5–24) | |
| CA125 ≥ 30 U ml$^{-1}$ | Normal morphology | [c] | 0.76 (0.09–5.96) | 35.6 (30–318) |
| | Abnormal morphology | 327 (150–708) | 119 (56–244) | 39.2 (30–>500) |
| | Abnormal morphology and volume ≥ 20 ml | 503 (218–1164) | 183 (83–402) | 39.9 (30–>500) |
| | Abnormal morphology and volume ≥ 50 ml | 617 (254–1495) | 224 (97–518) | 40.6 (30–134) |
| | Abnormal morphology and volume > 100 ml | 809 (302–2171) | 294 (114–756) | 40.6 (30–108) |

[a]95% confidence limits shown in parenthesis. [b]Previously published (Jacobs et al, 1996). [c]The figure could not be calculated because no index cancers occurred in this group until 2.3 years of follow-up

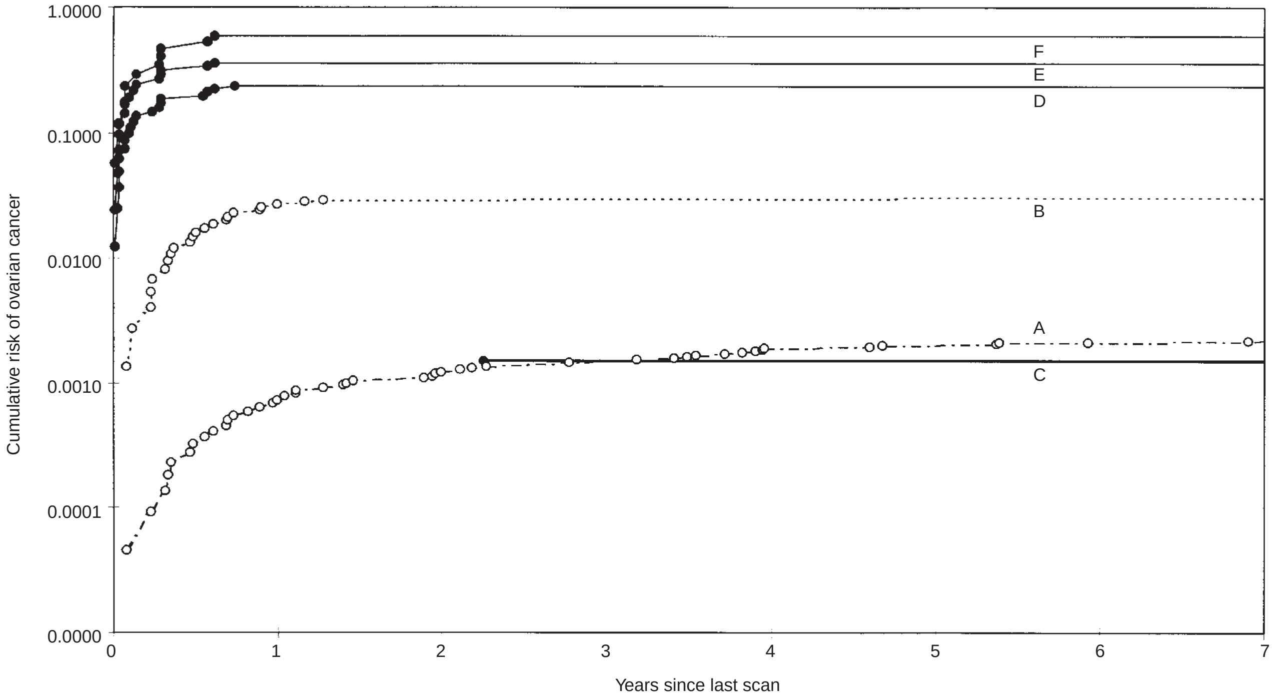

**Figure 1** Cumulative risk of developing an index cancer according to CA125 and ultrasound in asymptomatic post-menopausal women. (**A**) Entire study population of 22 000 women; (**B**) all women with a CA125 ≥ 30 U ml$^{-1}$; (**C**) CA125 ≥ 30 U ml$^{-1}$ and normal ovarian morphology; (**D**) CA125 ≥ 30 U ml$^{-1}$ and abnormal ovarian morphology; (**E**) CA125 ≥ 30 U ml$^{-1}$, abnormal morphology and ovarian volume ≥ 20 ml; (**F**) CA125 ≥ 30 U ml$^{-1}$, abnormal ovarian morphology and ovarian volume ≥ 100 ml. The cumulative risk curves (A,B) for all women with CA125 ≥ 30 U ml$^{-1}$ and the entire study population, shown in dotted lines, are reproduced from a previous analysis involving the same study population (Jacobs et al, 1996). Note that no index cancers developed in the women with a CA125 ≥ 30 U ml$^{-1}$ and normal ovarian morphology on scan in the initial 2 years following the scan episode. As a result, this curve (C) commences at 2.3 years the relative risk (RR) estimates were calculated using the Taylor series method (Rothman, 1986).

### RESULTS

Of the 22 000 study participants, 741 had a CA125 ≥ 30 U ml$^{-1}$ and underwent a scan. A further 26 volunteers with a CA125 ≥ 30 U ml$^{-1}$ declined an ultrasound ($n$ = 15) or were found to be premenopausal ($n$ = 11) and were therefore not included in the analysis. Twenty index cancers were diagnosed amongst the 741 women with a CA125 ≥ 30 U ml$^{-1}$ during a median follow-up of 6.8 years. The index cancers were invasive epithelial cancers of the following histological types; serous (seven), endometroid (six), clear cell (two), undifferentiated (one) and fallopian tube

© 1999 Cancer Research Campaign

(three). The number of index cancers at FIGO (International Federation of Gynaecology and Obstetrics) stages I, II, III and IV were seven, one, nine and two respectively. The histological type and precise stage were not known for one patient whose diagnosis was on the basis of imaging findings, clinical assessment and an ascitic tap which revealed adenocarcinoma cells.

A total of 662/741 women had normal ovarian morphology on scan of whom only one had a subsequent diagnosis of an index cancer (at 2.3 years of follow-up). Of the 79/741 women with abnormal ovarian morphology, 19 had a subsequent diagnosis of an index cancer. Of the women with abnormal morphology and an ovarian volume ≥ 20 ml and ≥ 100 ml, 15/41 and 10/17, respectively, had a subsequent diagnosis of an index cancer.

Figure 1 shows the relation between cumulative risk of developing an index cancer according to CA125 and ultrasound findings. Compared to the entire study population (curve A), women with a CA125 ≥ 30 U $ml^{-1}$ (curve B) had an increased risk of developing an index cancer. However, the subgroup of women with an elevated CA125 but normal ovarian morphology (curve C) had a similar cumulative risk to the entire study population (Jacobs et al, 1996) (0.15% vs 0.22%, 95% CI 0.02–1.12 vs 0.18–0.30). In contrast, the cumulative risk of developing index cancer in women with a CA125 ≥ 30 U $ml^{-1}$ and abnormal morphology (curve D) was 24% (95% CI 15–38). Further stratification of risk assessment was achieved by incorporating volume measurements in addition to morphology. The cumulative risk for index cancer in women with CA125 ≥ 30 U $ml^{-1}$ associated with abnormal morphology and ovarian volume of ≥ 20 ml (curve E) and ≥ 100 ml (curve F) was 37% (95% CI 22–61) and 59% (95% CI 32–111) respectively.

The RR of an index cancer at 1 and 5 years follow-up for various criteria are shown in Table 1. Among volunteers with a raised CA125 and abnormal ovarian morphology, the RR of index cancer at 1 year was 327 compared to the entire study population (RR 1.0). The highest RR at 1-year follow-up was for women with a CA125 ≥ 30 U $ml^{-1}$ whose ultrasound revealed abnormal morphology and ovarian volume ≥ 100 ml (RR 809, 95% CI 302–2165). Further stratification of women with abnormal ovarian morphology on the basis of CA125 level revealed that the 29 with CA125 > 50 U $ml^{-1}$ had a RR of 664 (95% CI 277–1590) and the ten with a CA125 > 100 U $ml^{-1}$ had a RR of 1682 (95% CI 486.5–5816).

### DISCUSSION

We have previously reported that asymptomatic post-menopausal women with a CA125 level ≥ 30 U $ml^{-1}$ have a 36-fold increased risk of ovarian cancer compared to the general population (Jacobs et al, 1996). This finding justified close surveillance of all post-menopausal women undergoing ovarian cancer screening who were found to have an elevated serum CA125. In the St Bartholomew's/Royal London Hospital Study this surveillance was undertaken using pelvic ultrasonography. The incidence of ovarian cancer on long-term follow-up and the details of ultrasound findings in the study were available for analysis. It was therefore possible to assess the impact of ultrasound on ovarian cancer risk stratification. The findings indicate that women at increased risk on the basis of an elevated CA125 can be further stratified for ovarian cancer risk according to ultrasound findings.

The majority (89%) of women with an elevated CA125 had normal scan findings. None of these women developed an index cancer within 1 year, and only 1/662 during the full duration of the study follow-up. This group of women were not at increased risk of an index cancer compared to the general population despite their CA125 elevation. On the basis of this finding, women with an elevated CA125 but normal ultrasound can be reassured about their level of risk of ovarian cancer. The protocol for our ongoing ovarian cancer screening trial reflects this data and women in this group are not recalled for additional surveillance.

In contrast, women with a raised CA125 and abnormal ultrasound findings are at a markedly increased risk of an index cancer. The risk of index cancer during the subsequent year for women with a CA125 ≥ 30 U $ml^{-1}$ and abnormal ovarian morphology on scan was 300-fold that of the entire population. This risk is significantly higher than the risk associated with an elevated CA125 alone (Jacobs et al, 1996). In view of the documentation of this high risk, the combination of CA125 elevation and abnormal ovarian scan findings justifies prompt investigation even when found in asymptomatic women. Our current screening protocol requires immediate referral for a specialist opinion for women in this category.

Further risk stratification was possible on the basis of ovarian volume measurements at ultrasound. Asymptomatic post-menopausal women with a CA125 ≥ 30 U $ml^{-1}$, abnormal ovarian morphology and an ovarian volume ≥ 100 ml were at 800-fold increased risk of diagnosis of an index cancer during the subsequent year. Risk stratification, according to the details of morphological findings may be valuable but was not feasible within the size of our dataset. Further analysis suggested that within the small group of women with abnormal ovarian morphology, the risk of ovarian cancer could be further categorized according to CA125 level.

The impact of screening for ovarian cancer on mortality is still uncertain. Furthermore, screening has a well-documented false-positive rate which can result in anxiety and morbidity associated with surgical investigation. For these reasons ovarian cancer screening should be limited to women at high risk because of a strong family history and women participating in research trials. Nevertheless, inappropriate screening is performed and clinicians are increasingly faced with the problem of CA125 elevation in asymptomatic women. The results of this analysis provide some guidance for management of post-menopausal women in this situation and for the design of future research trials to assess the impact of ovarian cancer screening. It is clear that ultrasound can separate asymptomatic post-menopausal women with raised CA125 levels into those who have normal scans and are not at increased risk of ovarian cancer and those with abnormal scans who are at substantially increased risk of ovarian cancer.

### ACKNOWLEDGEMENTS

We are grateful for the cooperation of British United Provident Association (BUPA) and the occupational health departments of Marks and Spencer and the National Westminster Bank. We thank R Woolas, AP Davies, J Bridges, I Stabile, T Fay, M Butcher and P Weidman for their contribution to the study as well as all the general practitioners whose collaboration was essential to the study. UM was funded by a Clinical Training Fellowship Grant from the St Bartholomew's Hospital Joint Research Board. ANR and NDM were funded by the charity Research into Ovarian Cancer (ROC). The trial was supported by grants from the ROC, GCRF, BMA (TP Gunton award) and Birthright (now Wellbeing).

 *© 1999 Cancer Research Campaign*

### REFERENCES

Campbell S, Bhan V, Royston P, Whitehead MI and Collins WP (1989) Transabdominal ultrasound screening for early ovarian cancer. *Br Med J* **299**: 1363–1367

Gadducci A, Zola P, Landoni F, Maggino T, Sartori E, Bergamino T and Cristofani R (1995*a*) Serum half-life of CA 125 during early chemotherapy as an independent prognostic variable for patients with advanced epithelial ovarian cancer: results of a multicentric Italian study. *Gynecol Oncol* **58**: 42

Jacobs IJ, Stabile I, Bridges J, Kemsley P, Reynolds C, Grudzinskas J and Oram D (1988) Multimodal approach to screening for ovarian cancer. *Lancet* **1**: 268–271

Jacobs IJ, Oram D, Fairbanks J, Turner J, Frost C and Grudzinskas JG (1990) A risk of malignancy index incorporating CA 125, ultrasound and menopausal status for the accurate preoperative diagnosis of ovarian cancer. *Br J Obstet Gynaecol* **97**: 922–929

Jacobs IJ, Davies AP, Bridges J, Stabile I, Fay T, Lower A, Grudzinskas JG and Oram D (1993) Prevalence screening for ovarian cancer in postmenopausal women by CA 125 measurement and ultrasonography. *Br Med J* **306**: 1030–1034

Jacobs IJ, Skates S, Davies AP, Woolas RP, Jeyerajah A, Weidemann P, Sibley K and Oram DH (1996) Risk of diagnosis of ovarian cancer after raised serum CA 125 concentration: a prospective cohort study. *Br Med J* **313**: 1355–1358

Nagele F, Petru E, Medl M, Kainz C, Graf AH and Sevelda P (1995) Preoperative CA 125: an independent prognostic factor in patients with stage I epithelial ovarian cancer. *Obstet Gynecol* **86**: 259–264

Rothman KJ (1986) *Modern Epidemiology*, pp. 218. Little Brown: Boston

Rustin GJ, Nelstrop AE, McClean P, Brady MF, McGuire WP, Hoskins WJ, Mitchell H and Lambert HE (1996*a*) Defining response of ovarian carcinoma to initial chemotherapy according to serum CA 125. *J Clin Oncol* **14**: 1545–1551

Rustin GJ, Nelstrop AE, Tuxen MK and Lambert HE (1996*b*) Defining progression of ovarian carcinoma during follow-up according to CA125: a North Thames Ovary Group Study. *Ann Oncol* **7**: 361–364

Tingulstad S, Hagen B, Skjeldestad FE, Onsrud M, Kiserud T, Halvorsen T and Nustad K (1996) Evaluation of a risk of malignancy index based on serum CA125, ultrasound findings and menopausal status in the pre-operative diagnosis of pelvic masses. *Br J Obstet Gynaecol* **103**: 826–831

*© 1999 Cancer Research Campaign* 
